# Fate of the soil seed bank of giant ragweed and its significance in preventing and controlling its invasion in grasslands

**DOI:** 10.1002/ece3.6238

**Published:** 2020-04-12

**Authors:** Hegan Dong, Tong Liu, Zhongquan Liu, Zhanli Song

**Affiliations:** ^1^ College of Life Science Shihezi University Shihezi China; ^2^ Rural Energy and Environment Work Station in Yili Yining Xingjiang China; ^3^ Yili Vocational and Technical College Yining China; ^4^ Yili State Forestry Academy Yining China

**Keywords:** *Ambrosia trifida* L., biological invasions, invasive species management, reduced soil seed bank density, seed consumption, seed life span, soil seed bank

## Abstract

Giant ragweed (*Ambrosia trifida*, L. henceforth referred to as GR), an annual non‐native invasive weed, may cause health problems and can reduce agricultural productivity. Chemical control of GR in grasslands may have irreversible side effects on herbs and livestock. In an attempt to propose a solution to the harmful effects of GR on grasslands, this study explores the fate of its soil seed bank (SSB) and considers the physical control of its SSB reduction.

By studying GR distributed in grasslands of the Yili Valley, Xinjiang, China, we measured the spatial and temporal changes in seed density, seed germination, dormancy, and death. We analyzed seed germination, dormancy, and death following different storage periods. The study analyzed population characteristics over time, including seed fate, and examined physical control methods for reducing the SSB density.

The SSB of GR occurs in the upper 0–15 cm of soil in grasslands. Seed density in the SSB decreased by 68.1% to 82.01% from the reproductive growth period to the senescence period. More than 98.7% of the seeds were rotten, eaten, germinated, dispersed, or died within one year after being produced. The seed germination rate of the SSB decreased with the number of years after invasion. When stored for 0.5 or 3.5 years, seed germination rates fell by 40%, during which time seed death rate increased by almost 40%. When GR was completely eradicated for two consecutive years, the SSB and population densities decreased by >99%.

The vast majority of GR seeds germinated or died within one year; the germination rate decreased significantly if the seeds were stored dry at room temperature for a long time. Newly produced seeds are the main source of seeds in the SSB. Therefore, thoroughly eradicating GR plants for several years before the seeds can mature provides an effective control method in grasslands.

## Graphical abstract

Pictures A and B were taken on June 3, 2018 and July 24, 2019, respectively, during the vegetative growth period in Yili Vally. Pictures A and B, respectively, show the distribution of GR in grassland from large and small perspectives and the green areas indicated by the arrow are all GR.

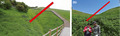

## 1 INTRODUCTION

Giant ragweed (*Ambrosia trifida* L. GR) is a natural colonizer of disturbed areas native to North America (Goplen, 2015); its pollen creates a threat to human health as a major cause of hay fever (Abul‐fatih & Bazzaz, 1979; Hamaoui‐Laguel et al., 2015; Richter et al., 2013). The distribution of GR and the effects of its pollen are expected to become increasingly serious under global warming (Rasmussen, Thyrring, Muscarella, & Borchsenius, 2017). GR also causes crop reduction and other forms of agricultural loss (Harrison, Regnier, & Schmoll, 2003; Kong, Wang, & Xu, 2007; Schutte et al., 2008).

Grasslands are often rich in plant diversity, supporting both grasses and nongrasses that may be annual, biennial, or perennial plants. GR often readily invades various types of grasslands (Dukes, 2001). The control of GR in grasslands is more complicated than in farmland, because the simple use of chemical herbicides to control GR will harm both herbs and livestock. Meanwhile, the chemical control of GR is difficult due to evolved resistance to acetolactate synthase‐inhibiting herbicides and glyphosate (Ganie et al., 2017; Harre et al., 2017; Heap, 2014). Therefore, it is necessary to search for a more reasonable and safer way to control GR.

Giant ragweed propagates exclusively by seeds (Rasmussen et al., 2017). This annual plant uses an r‐strategy during reproduction, featuring rapid development, a large proportion of reproductive allocation, a short generation cycle, and production of a large number of individuals. GR, like common ragweed, also employs a bridgehead invasion strategy (Boheemen, Lombaert, Nurkowski, Gauffre, & Hodgins, 2017), so that the SSB plays an important role in population maintenance and growth. Reducing the persistence of the SSB is an important goal for weed management systems (Davis, 2006; Davis, Dixon, & Liebman, 2004; Goplen et al., 2017; Krinke et al., 2005; Mourik, Stomph, & Murdoch, 2005).

Giant ragweed produces several empty, nonviable seeds that deter seed predators by increasing foraging time, thereby increasing the survival rate of the viable seeds (Goplen, 2015). Seed predation by rodents and invertebrates has been shown to remove as many as 88% of GR seeds in one year in no‐tillage corn cultivation (Harrison et al., 2003). Replenishment of the SSB of GR can be controlled by adjusting the cultivation methods in farmland (Goplen, 2015; Goplen et al., 2016; Page & Nurse, 2015). Grasslands are typically not plowed, and as a result, feature abundant biodiversity. However, in our literature search, we found no studies of GR control in grasslands.

Seeds have a limited lifespan, and their ability to germinate varies with storage time. Although most studies have shown that the seed lifespan of GR is less than four years (Goplen, 2015; Stoller & Wax, 1973), some seeds were viable after nine years of burial (Harrison, Regnier, Schmoll, & Harrison, 2007), and a small number of GR seeds may live for more than 15 years (Hartnett, Hartnett, & Bazzaz, 1987). Moreover, the environment affects the seed lifespan (Probert, Daws, & Hay, 2009) via water, oxygen, temperature, and light (Finch‐Savage & Leubner‐Metzger, 2006). Grassland and farmland ecosystems are quite different, and studying the seed lifespan of GR in grasslands compared with that in farmland deserves further investigation.

Seed lifespan affects the ability of seeds in the SSB to germinate (Deveny & Fox, 2006). Plant species with a persistent seed bank can spread germination over time, thus allowing some seeds to germinate when appropriate conditions exist; this serves as an efficient mechanism to mitigate the hazardous effects of severe environmental conditions (Fletcher et al., 2015). Because of this, it is meaningful to research the germination characteristics of GR seeds in the SSB during different invasion years and to consider possible physical controls for SSB reduction.

**FIGURE 1:**
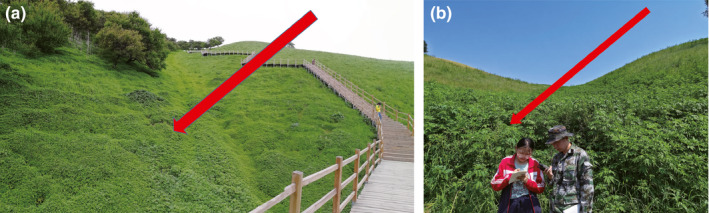
Damage caused by giant ragweed (*Ambrosia trifida* L.) in grassland. Pictures a and b were taken on June 3, 2018 and July 24, 2019, respectively, during the vegetative growth period in Yili Vally. Pictures a and b show the respective distributions of GR in a grassland from large and small perspectives, and the green areas indicated by the arrows are all GR

Giant ragweed invaded the Yili Valley in Xinjiang, China in 2010; the distribution area had increased by 2,150 times by 2016 (Figure 1). The main reason is large‐scale dispersion assisted by cattle and sheep combined with the topography of the surrounding hillsides; these factors are not easy to control and manage effectively (Dong et al., 2017). This study considers whether we can prevent the introduction of GR and control GR by reducing the SSB density via physical methods.

The seed fate in this study refers to the characteristics of SSB density, seed germination, seed dormancy, and seed death in different soil layers and during different years after the invasion of GR. We carried out a prevention and control project designed to reduce the seed numbers in the SSB. We hypothesized that the SSB density would vary significantly by soil depth and over time after invasion; we further hypothesized that invasions of GR could be controlled by reducing the density of seeds in the SSB in grasslands. We addressed four questions in this study: (1) What is the temporal and spatial variation of SSB density in grasslands? (2) What dynamic changes in seed density occur during different years after invasion in grasslands? (3) What are the characteristics of seed germination, seed dormancy, and seed death in the SSB in different years and following different periods of storage? (4) Can we control GR more effectively by reducing the SSB density in grasslands?

## 2 MATERIALS AND METHODS

### 2.1 Research area

**FIGURE 2:**
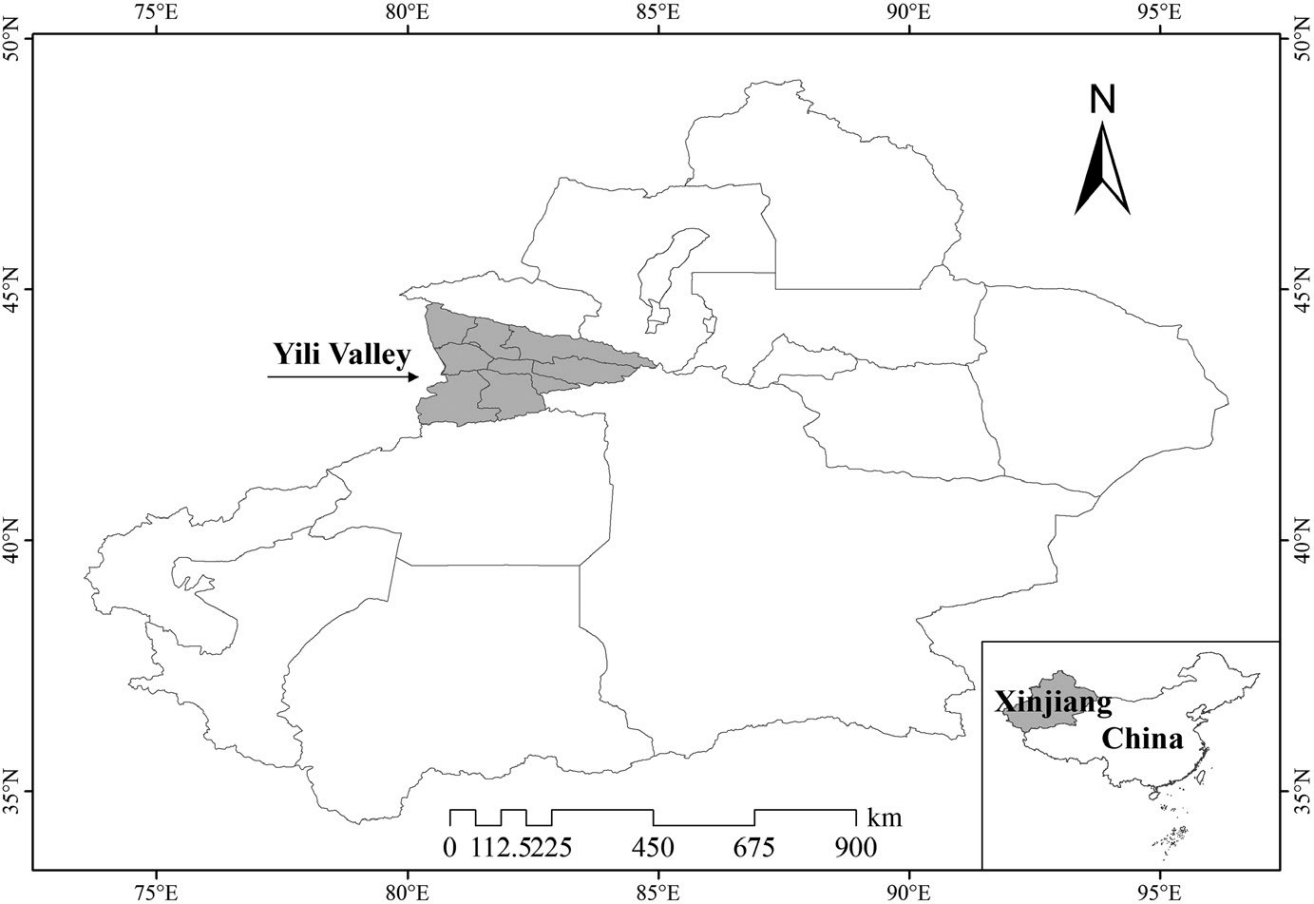
Location of the Yili Valley study area within the Xinjiang Autonomous Region

The study area is located in the Yili Valley (42°14′16′′–44°53′30′′N, 80°09′42′′–84°56′50′′E) of the Xinjiang Autonomous Region (Figure 2), with an average annual temperature of 10.4°C and an average annual precipitation of 417.6 mm. The altitude of the GR distribution area in grasslands is 900–1,400 m. GR occurs mainly in grasslands where fruit trees are abundant, in a region known as the “world's apple source species” and the “Chinese wild fruit gene pool” (Chen, 1993). Hills are the main topography, and wild apricot (*Armeniaca vulgaris*) and wild apple (*Malus sieversii*) are scattered throughout the valley. The grassland is mainly for grazing (Figure 1). More than 2,000 hectares of GR distribution area in 2017 and the high density of GR had posed a serious threat to the seedling regeneration of wild apricots and apples, resulting in forage yield reduction (Dong et al., 2017).

### 2.2 Experimental designs and methods

#### 2.2.1 Experiment 1. SSB collection and determination

##### Soil physical and chemical properties

Surface grassland soil (0–20 cm depth) was divided into two layers. The soil in each 10 cm layer was sampled in July 2017, and soil properties were determined as follows. Total nitrogen, total phosphorus, and total potassium were determined using the micro‐Kjeldahl, sodium hydroxide melting‐molybdenum, anti‐colorimetric, and flame photometry methods, respectively. Soil pH was measured using a Mettler‐Toledo pH meter (UB‐10), and soil conductivity was measured using a conductivity meter (HACH). Soil organic matter content was checked using the K_2_CrO_7_‐H_2_SO_4_ external heating method. Alkaline hydrolysis nitrogen, available P, and available K were measured using the alkaline hydrolysis diffusion method, Mo‐Sb colorimetry, and the ammonium acetate method, respectively. Six sampling points were collected (more than 1 km apart) randomly in grasslands of the study area, and a total of 12 soil samples were analyzed.

##### Distribution depth of SSB

To determine the depth limit for GR seeds distributed in grasslands, three GR populations (more than 1 km apart) with the longest invasion time (all populations being at 6 years after invasion based on our fixed‐point observations) were selected on April 3, 2018, and in each population three replicate soil samples were taken. Each 20 cm × 20 cm sampling point was divided into five soil layers: 0–5, 5–10, 10–15, 15–20, and 20–25 cm; and a total of 3 (population) × 3 (repetition) × 5 (soil layer) resulted in 45 soil samples being made. No GR seeds were observed in soil below 15 cm (Table 1). Therefore, the SSB of each year collected in this experiment was obtained from the 0–15 cm soil layer.

**TABLE 1:** Soil seed bank (SSB) density of giant ragweed (*Ambrosia trifida* L.) at 6 years after invasion

Soil depth/cm	0–5	5–10	10–15	15–20	20–25
SSB density (grains/m^2^)	9,345 ± 51.4a	752 ± 8.3b	439 ± 8.1c	0d	0d

Note

Different letters indicate significant differences (*p* < .05) using a least significant difference test.

##### Collection of SSB

Soil samples were collected in four seasons during 2018: germination (April 10), vegetative growth (June 10), reproductive growth (September 10), and senescence (November 10). Based on the date of invasion, eighteen GR populations (more than 1 km apart) were collected at 1, 2, 3, 4, 5, and 6 years after invasion. Soils were collected from each sampled population, and seeds of GR were counted. Each 20 cm × 20 cm sampling point was divided into four soil layers: 0–2 cm, 2–5 cm, 5–10 cm, and 10–15 cm, for a total of 6 (year after invasion) × 4 (season) × 3 (population) × 4 (soil layer) = 288 soil samples being analyzed.

##### Determination of SSB density

Seeds of GR can be up to 11 mm wide and 14 mm long and are substantially larger than seeds of most annual weed species (Bassett & Crompton, 1982; Sako et al., 2001); this makes it easy to separate the seeds from soil by washing in water. Soil samples were collected in plastic bags for laboratory work. The bags were opened immediately for ventilation, and then the samples were washed for three days. Soil samples were placed in a plastic bucket (40 cm diameter; 60 cm height), and enough water was added to soak the soil for 12 hr. Then, samples were thoroughly stirred and sieved using a 2 mm sieve; the sieve was rinsed continuously with water until the soil was completely removed, leaving only seeds, pebbles, and other granular impurities. Next, the seeds of GR were removed by hand and counted.

#### 2.2.2 Experiment 2. Dynamic changes in GR seed density from 1 to 6 years after invasion

In this study, the SSB density in each reproductive growth period and the density of newly produced seeds from plants of the current population were taken as the starting points, and the density of SSB during the reproductive growth period in the next year was taken as the end point of the analysis.

1. Starting seed density = newly produced seed density (seed quantity per plant × population density) + SSB density in the reproductive growth period.
2. End point seed density = SSB density in the reproductive growth period of the next year.
3. Reduction of nonempty seed density from reproductive growth to senescence period = Starting seed density – empty seed density – SSB density in the senescence period. The decrease in seed density from reproductive growth to the senescence period mainly includes the loss of empty seeds, animal/invertebrate grazing, and decay of nonempty seeds. The density of empty seeds can be counted (empty seeds per plant × population density).
4. Reduction of seed density in overwintering = SSB density in the senescence period – SSB density in the germination period of the next year.
5. Reduction of seed density during the germination period (except germination) = SSB density in the germination period of the next year – SSB density in the vegetative period of the next year – density of germinated seeds in the next year. Reduction of seed density from germination to the vegetative growth period includes seed germination, decay, animal/invertebrate grazing, and outward diffusion.
6. Reduction of seed density during the vegetative growth period = SSB density in the vegetative growth period of the next year – SSB density in the reproductive growth period of the next year.

#### 2.2.3 Experiment 3: Determination of seed germination, seed dormancy, and seed death in the SSB for different invasion years

Before the seed germination in April 2018, the SSB density defined the population density in that year, so the SSB in the germination period was chosen for experiments on seed germination, dormancy, and death characteristics.

##### Germination rate

SSB seeds were collected as experimental seeds in April 2018. No seeds were observed in the 2–5 cm, 5–10 cm. or 10–15 cm soil layers invaded for one year, nor in the 10–15 cm layer invaded for two years. The seeds from the 0–2 cm soil layer that had been invaded for one year, the 5–10 cm soil layer invaded for up to two years, and the 10–15 cm soil layer invaded for up to three years were insufficient in numbers for germination experiments. Therefore, an additional 100 × 100 cm soil sample was added to each population in the three cases mentioned above when collecting soil on April 13 to ensure that the seed quantity of each population with seeds in each of these soil layers was greater than 30.

Each group of seeds was placed in a Petri dish. The germination rate of the seeds was determined at (20/10)°C, 12 hr/12 hr light/darkness and 3,000 lux light intensity (Liu et al., 2019). An appropriate amount of water was added daily to each Petri dish to keep the filter paper wet. The experiment lasted for 60 days. When no seeds germinated in a single Petri dish for five consecutive days, this suggested the end of germination. Each experiment was conducted with 30 randomly selected seeds and was repeated six times with a total of 6 (year after invasion) × 6 (repetition) × 4 (soil layer) resulting in 144 samples.

The germination rate = number of germinated seeds/30 × 100%.

##### Dormancy rate and death rate

The nongerminating seeds in tested dishes were collected, and viability was determined by the 2,3,5 triphenol tetrazolium chloride method (Liu et al., 2019). Viable but nongerminated seeds were classified as dormant.

Thedormancyrate=numberofdormantseeds/30×100%.

Thedeathrate=numberofdeadseeds/30×100%.

#### 2.2.4 Experiment 4: Determination of seed germination, seed dormancy, and seed death following different periods of dry storage

In 2015 and during the following three years, we collected a large number of mature GR seeds from more than 100 plants in the GR core distribution area of the grassland. The seeds were initially placed in the laboratory with dry storage at room temperature (10–25°C). In April 2019, seeds stored for 0.5, 1.5, 2.5, and 3.5 years were sampled for germination, dormancy, and death experiments. Each experiment was conducted with 30 randomly selected seeds and was repeated six times; a total of 4 (storage year) × 6 (repetition) resulted in 24 samples. The experimental design and statistical methods of analysis for seed germination rate, seed dormancy rate, and seed death rate were the same as in Experiment 3.

#### 2.2.5 Experiment 5: Determination of population density and seed yield

To analyze changes in population density and seed yield during different periods of invasion, eighteen 20 m × 20 m populations (more than 1 km apart) of GR that had invaded 1, 2, 3, 4, 5, and 6 years ago were selected, and the following indicators were counted for each group.

##### Population density in the reproductive growth period

The number of GR plants was counted using three 2 m × 2 m square areas in September 2018. Three samples were taken for each population, and a total of 6 (year after invasion) × 3 (population) × 3 (repetition) resulted in 54 samples.

##### Seed quantity per unit area

In October 2018, three 2 m × 2 m samples were selected from different populations, and six GR plants were randomly selected from each sample; all seeds on the plants were harvested to count the total numbers of filled seeds and empty seeds. Seed quantity per unit area = Seed number per plant × population density. Empty seed quantity per unit area = Empty seed number per plant × population density. Three samples were taken for each population, and a total of 54 samples were taken [6 (year after invasion) × 3 (population) × 3 (repetition), resulting in 54 samples].

#### 2.2.6 Experiment 6: Effect of physical control on reducing seed bank and population densities

Targeted at testing the physical control effects on reducing the SSB density, three GR populations with four years of invasion were selected in the grasslands in April 2016. A 50 m × 50 m sample plot was enclosed in a fence in each population. During the seed dispersal period (September to April), we covered the sample plot with transparent gauze to prevent external seeds from falling into the plot. During the reproductive growth period, all GR plants in each sample plot were eradicated in 2016 and 2017, so that no new seeds were produced for two consecutive years in each plot. The SSB densities in April and the population densities in August 2016, 2017, and 2018 were sampled and measured. Specific sampling methods were the same as in Experiments 1 and 5. A total of 3 (year) × 3 (population) × 3 (repetition) × 4 (soil layer) resulted in 108 soil samples. A total of 3 (year) × 3 (population) × 3 (repetition) resulted in 27 density samples.

### 2.3 Statistical analysis

**FIGURE 3:**
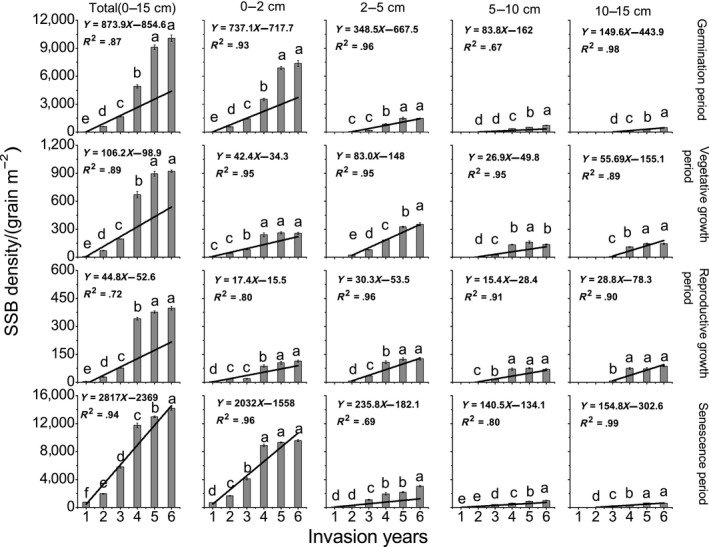
Spatial and temporal variation of soil seed bank (SSB) density of giant ragweed (*Ambrosia trifida* L.) in grassland. Different letters indicate significant differences between different invasion years (*p* < .05) using a least significant difference test

**FIGURE 4:**
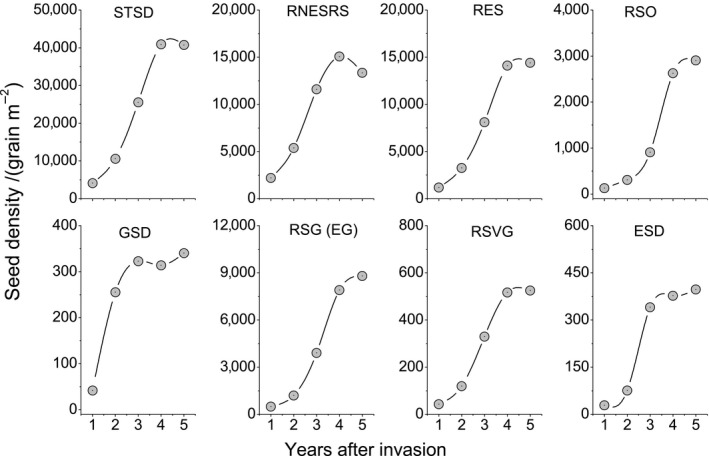
Dynamic change characteristics of seed density of giant ragweed (*Ambrosia trifida* L.) in grasslands. Note: STSD, starting seed density; RNESRS, reduction of nonempty seed density from the reproductive growth to senescence periods; RES, reduction of empty seeds; RSO, reduction of seed density in overwintering; GSD, germinated seed density; RSG (EG), reduction of seed density during the germination period (except germination); RSVG, reduction of seed density during vegetative growth period; ESD, end point seed density.

**FIGURE 5:**
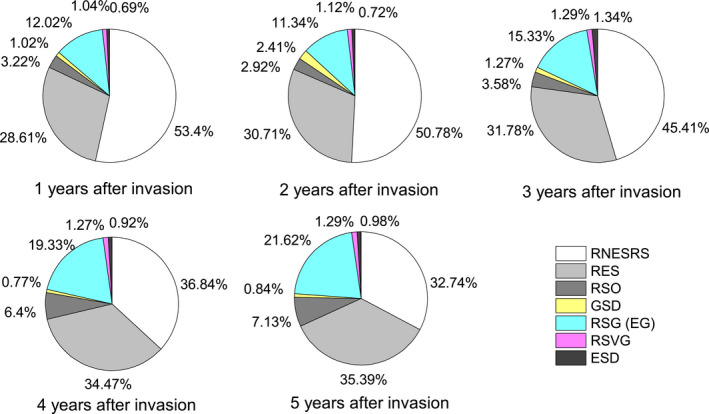
Proportions of seed density of giant ragweed (*Ambrosia trifida* L.) in each stage to starting seed density. Note: STSD, RNESRS, RES, RSO, GSD, RSG (EG), RSVG, and ESD are defined in Figure 4

**FIGURE 6:**
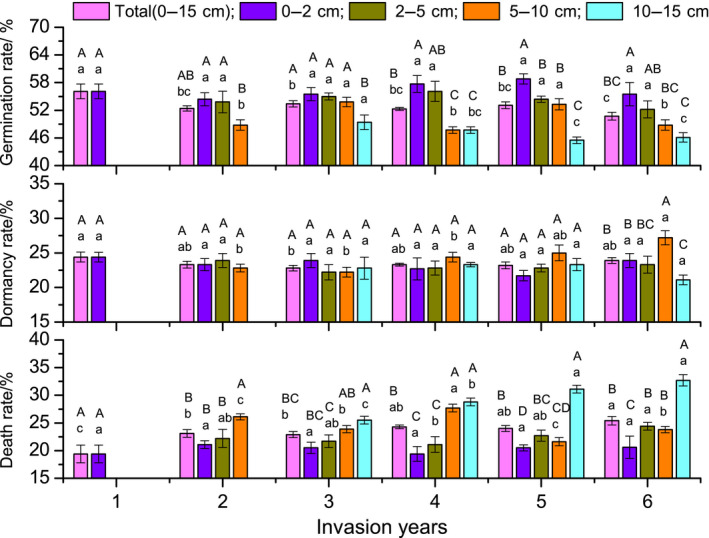
Seed germination, dormancy, and death rate of giant ragweed (*Ambrosia trifida* L.) in the soil seed bank (SSB) during the germination period at different years after invasion. Different lowercase and uppercase letters represent significant differences between different invasion years in the same soil layer and differences between different soil layers in the same invasion year, respectively (*p* < .05), using a least significant difference test

**FIGURE 7:**
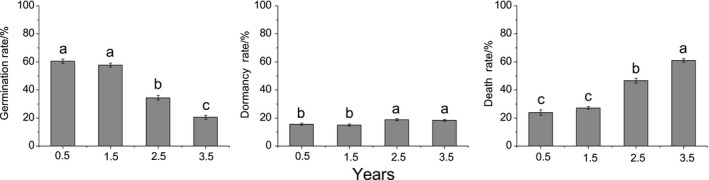
Seed germination, dormancy, and death rate of giant ragweed (*Ambrosia trifida* L.) following different periods of storage. Different letters indicate significant differences between different storage years at *p* < .05 using a least significant difference test

**FIGURE 8:**
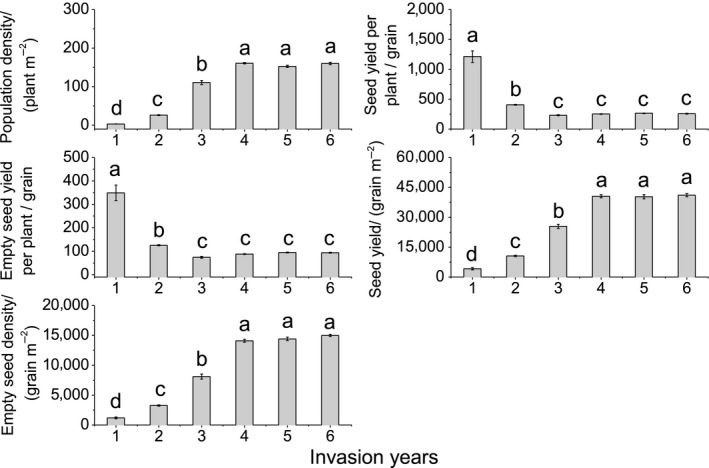
Population density and seed yield of giant ragweed (*Ambrosia trifida* L.) at different years after invasion. Different letters indicate significant differences between different invasion years (*p* < .05) using a least significant difference test

Data are expressed as means ± *SE*. One‐way ANOVA analyses and least significant difference multiple comparisons were used to explore the differences in soil physical and chemical properties (Table 2), SSB density (Tables 1 and 3; and Figure 3), population density (Table 3), seed germination rate, dormancy rate and death rate (Figures 6 and 7) and population density and seed yield (Figure 8). One‐way ANOVA analyses were used to explore the differences in SSB density between six years, and linear regression analyses were used to analyze the trend of SSB with years (Figure 3). Trend and pie chart analyses were used to explore the dynamic change characteristics of seed density (Figures 4 and 5). IBM SPSS statistics 20 was used for data analysis, and OriginPro 8.5 was employed for graphics.

**TABLE 2:** Soil physical and chemical properties of grassland in Yili Valley, Xinjiang Autonomous Region, China

Soil (cm)	Total nitrogen (%)	Total phosphorus (g/kg)	Total potassium (g/kg)	Available nitrogen (mg/kg)	Available phosphorus (mg/kg)	Available potassium (mg/kg)	Organic matter (%)	pH	Conductivity (us/m)
0–10	13.3 ± 2.01a	0.0768 ± 0.0067b	22.3 ± 4.27a	2.0 ± 0.66a	0.0631 ± 0.0088a	14.4 ± 2.62a	16.9 ± 2.62a	7.93 ± 0.93a	11.9 ± 0.17a
10–20	9.63 ± 1.67b	0.111 ± 0.014a	24.9 ± 6.21a	1.9 ± 0.46a	0.0285 ± 0.0031b	11.9 ± 1.97a	16.9 ± 1.27a	7.98 ± 0.62a	11.8 ± 0.42a

Note

Different letters indicate significant differences between soil layers (*p* < .05) using a least significant difference test.

**TABLE 3:** Soil seed bank (SSB) density and population density of giant ragweed (*Ambrosia trifida* L.) at different treatment times

Year	Treatment time/year	Soil seed density/(grain/m^2^)					Population density/(plant/m^2^)
		0–15 cm	0–2 cm	2–5 cm	5–10 cm	10–15 cm	
2016	0	4,877 ± 61.72a	3,487 ± 48.8a	863.3 ± 16.0a	356.8 ± 10.1a	169.1 ± 3.68a	160.2 ± 3.1a
2017	1	117.7 ± 2.69b	34.6 ± 1.07b	32.0 ± 1.1b	28.3 ± 1.18b	24.6 ± 0.99b	7.0 ± 0.41b
2018	2	31.9 ± 1.10b	2.44 ± 0.24b	5.89 ± 0.48b	11.0 ± 0.47c	12.5 ± 0.44c	0.33 ± 0.17c

Note

Different letters indicate significant differences (*p* < .05) using a least significant difference test.

## 3 RESULTS

### 3.1 Spatial and temporal variation of SSB density

After comparing the soil physical and chemical properties of the 0–10 cm and 10–20 cm soil layers, there were no significance differences in total phosphorus, total potassium, available nitrogen, available potassium, organic matter, pH, or conductivity. Total nitrogen and available phosphorus in the 0–10 cm soil layer were significantly higher than those in the 10–20 cm layer, and total phosphorus in the 0–10 cm soil layer was significantly lower than that in the 10–20 cm layer (Table 2).

With the increasing number of years after invasion, the total SSB density and the SSB density in each soil layer during germination, vegetative growth, reproductive growth, and senescence periods all increased significantly (Figure 3). Seeds were mostly distributed in the 0–5 cm soil layer, in which the proportions of SSB seeds for 6 years of invasion were 88%–100% in the germination period, 63%–100% in the vegetative growth period, 57%–100% in the reproductive growth period, and 88%–98% in the senescence period. Compared with different periods, the SSB density in the senescence period was greater than in other periods. The SSB density in the senescence period was 35.7 times higher than that in the reproductive growth period at 6 years after invasion (Figure 3).

With the increasing number of years after invasion, seeds were found at deeper soil depths. In the first year after invasion, seeds were found only in the 0–2 cm soil layer in the germination, vegetative growth, and reproductive growth periods, but were found in the 0–10 cm soil layer in the senescence period. In the second year after invasion, seeds were distributed in the 0–15 cm soil layer in the senescence period, indicating that after GR had invaded the grassland for 1–2 years, a deeper SSB could be formed (Figure 3).

The SSB density in the 0–2 cm soil layer and 0–15 cm soil layer tended to be stable, and SSB density had not increased significantly by the sixth year after invasion. Seeds in the 0–2 cm soil layer were the main source of seed germination, so the germination ability of the population tended to be stable by the sixth year after invasion, while the SSB densities in the 2–5 cm, 5–10 cm, and 10–15 cm soil layers remained unstable, and the seeds tended to diffuse to the 5–15 cm nongerminating soil layer (Figure 3).

### 3.2 Dynamic change characteristics of seed density

With the increasing number of years after invasion, the density of starting seed density, SSB density, seed germination as well as the reduction of seed density in all periods significantly increased (Figure 4). The most important period of seed reduction was from reproductive to senescence, when the proportion of lost seeds in the starting seed densities during 1–5 years after invasion were 82.01%, 81.49%, 77.19%, 71.29%, and 68.13%, respectively. Seeds for germination in the next year accounted for less than 2.41% of the starting seed density. Seeds forming the SSB of the senescence period in the next year accounted for less than 1.34% of the starting seed density (Figure 5).

### 3.3 Seed germination, dormancy, and death in the SSB in the germination period with increasing years after invasion

With an increasing number of years after invasion, the seed germination rate showed a downward trend. The total seed germination rates of the SSB at 1 and 6 years after invasion were 56.1% and 50.7%, respectively, representing a significant decrease of 5.4%. With an increasing number of years after invasion, the seed death rate showed an upward trend. The total seed death rates of the SSB at 1 and 6 years after invasion were 19.4% and 25.4%, respectively, representing a significant increase of 6% (Figure 6).

With the increase of soil depth, the seed germination rate showed a downward trend; the germination rates in the 0–2 cm and 10–15 cm soil layers were 55.5% and 46.1%, respectively, a decrease of 9.4% in the sixth year after invasion. The seed death rate showed an upward trend over time; the seed death rates in the 0–2 cm and 10–15 cm soil layers were 20.6% and 32.7%, respectively, an increase of 12.1% in the sixth year after invasion (Figure 6).

### 3.4 Seed germination, dormancy, and death following different periods of storage

After 2.5 years of storage, the germination rate of GR seeds began to decrease significantly; meanwhile, the dormancy and death rates increased significantly (Figure 7). In addition, after 2.5 years of storage, the germination and death rates were 34.44% and 46.66%, although the germination rate fell more than the death rate. When seeds were stored for 0.5 or 3.5 years, the germination rates were 61% and 21%; additionally, the dormancy rates increased from 16% to 18%, and the death rate increased from 24% to 61% (Figure 7).

### 3.5 Population density and seed yield in different years after invasion

With the increasing number of years after invasion, population density in the reproductive growth period, seed yield per unit area, and empty seed yield per unit area all increased significantly (Figure 8). The respective indicators at the fourth year after invasion were 48.7, 9.95, and 12.7 times higher than those at the first year after invasion. By the fifth year after invasion, those population density and seed yield indicators had stabilized. However, with the increasing number of years after invasion, seed yield per plant and empty seed yield per plant all decreased significantly. The respective indicators at the first year after invasion were 5.21 and 4.71 times higher than those at the third year after invasion. By the fourth year after invasion, the per plant seed yield indicators had stabilized (Figure 8).

### 3.6 Effects of physical control on reducing seed bank and population densities

In 2017, after GR had been completely eradicated for one year, SSB density in all soil layers decreased significantly (Table 3). The closer the SSB was to the ground surface, the more the SSB density decreased. Compared with 2016, the SSB density of the 0–2 cm soil layer decreased by 99.0%, and that of the 10–15 cm soil layer decreased by 92.6%. In 2018, the SSB had decreased by 99.34% (Table 3).

The population density of GR decreased significantly over time with plant removal. Compared with 2016, the population density decreased by 99.79% in 2018 (Table 3).

## 4 DISCUSSION

The SSB of GR only occurs in the upper 0–15 cm of soil in grasslands and is mostly distributed in the upper 0–5 cm of the soil. The maximum SSB occurs in the senescence period. The highest SSB density in grasslands was 14,200 grain m^−2^ at six years after invasion (Figure 3), which is much higher than the SSB in farmlands (Goplen, 2015; Goplen et al., 2016; Page & Nurse, 2015).

Population density and seed yield of GR were previously found to be highly correlated (Harrison, Regnier, Schmoll, & Webb, 2001). Population density of GR and seed yield per unit area stabilized and did not increase significantly by five years after invasion (Figure 8). The total SSB density had not increased significantly by six years after invasion. The SSB density had become more uniform by six years after invasion (Figure 3). Persistence of GR populations appears to depend on frequent inputs to its seed bank that offset limitations imposed by high seed predation and rapid demise of its active seed bank (Harrison et al., 2007). Stable population density is the foundation of a stable SSB of GR. However, the SSB density at relatively deeper soil (5–15 cm) increased continuously during six years after invasion; this played an important role in the stability and persistence of the population.

With an increasing number of years after invasion, the numbers of total seeds and reduced seeds in each period increased significantly (Figure 4). A single GR plant can produce 3,000 to 5,000 seeds/m^2^ in an ideal environment (Abul‐Fatih & Bazzaz, 1979; Harrison et al., 2001). However, our research revealed that the maximum seed yield of GR can reach 41,100 m^−2^ (Figure 8). We conclude that GR plants can produce a massive amount of seed yield, and that the damage caused by GR in grasslands is very serious. In areas where GR is distributed, the weed will become the dominant species, seriously endangering the grassland biodiversity and forage yield (Figure 2). Weed seeds may germinate and emerge or die, while fungi and other soil microorganisms may cause seeds to decay, and seed predators such as birds and rodents may consume the seeds (Buhler, King, Swinton, Gunsolus, & Forcella, 1997; Chee‐Sanford, Williams, Davis, & Sims, 2006; Kremer, 1993; Myers & Harms, 2010). The seeds of GR have a high nutritional value that offers an important food source for rodent and invertebrate populations (Harrison et al., 2003). A single GR plant produces many empty seeds, most of which will be eaten or will decay in the year in which they are produced (Goplen, 2015). GR seed can be depleted relatively quickly, with up to 50% of the seeds being consumed during one overwintering period in farmland (Harrison et al., 2003; Regnier et al., 2008). Our research shows that seeds of GR decreased by 68.1% to 82.01% from the reproductive growth period to the senescence period (Figure 5). Nordby, Williams, and Chee‐Sandford (2005) reported that seedling emergence accounted for 5 to 29% of total seed losses in farmland. According to our research, germinated seeds accounted for less than 2.41% of the number of starting seeds (Figure 5). Seed density dynamic change characteristics of GR in grassland are different from those in farmland. Seeds that formed the seed bank in the reproductive growth period in the next year accounted for less than 1.34% of the number of starting seeds (Figure 5). Overall, more than 98.66% of the seeds were exhausted in one year. Therefore, the SSB density of GR in grassland is determined by the number of seeds produced in that year, not by the accumulation of seeds over several years.

The seed germination rate of the SBB decreased with the increasing number of years after invasion and with soil depth. Seed death rate showed an opposite trend, but there was no significant difference in the 0–2 cm soil layer over time (Figure 6). According to Harrison et al. (2007), rates of seed demise were inversely proportional to burial depth, while our study obtained the opposite result. With increasing years after invasion and deeper soil, a larger proportion of seeds were stored for a longer time. The proportion of “old seeds” in deep soil is higher, so the germination rate is lower. However, the total seed germination rate decreased insignificantly, mainly because the number of newly produced seeds was higher.

When seeds were stored for 2.5 years, the germination rate began to decrease significantly, and the death rate increased sharply (Figure 7). The germination and dormancy rates of GR seeds were still 20.55% and 18.33% after 3.5 years of storage. According to Stoller and Wax (1973), the seed lifespan of GR is less than four years. In addition to its lifespan, seed vigor is closely related to animal feeding, pathogenic microorganism infection, and depth of burial (Harrison et al., 2007) as well as to soil temperature, moisture, oxygen, salinity, other environmental factors (Silvertown & Charlesworth, 2001), and agronomic practices (Clements et al., 2004). A diverse range of dormancy mechanisms has evolved in keeping with the diversity of climates and habitats in which plants operate (Finch‐Savage & Leubner‐Metzger, 2006). These are the reasons why the death rate of GR seeds dry stored is lower in grasslands.

Dormancy has a very wide biogeographical distribution (Baskin & Baskin, 1998; Fenner & Thompson, 2005). We found that the dormancy rate of GR seeds could reach 18.33% if stored for 3.5 years (Figure 7), which could greatly promote the maintenance of the population.

Giant ragweed's active seed bank is relatively short‐lived when the soil is left undisturbed and no new seed inputs are allowed (Abul‐Fatih & Bazzaz, 1979; Nordby et al., 2005; Stoller & Wax, 1974). In the present study, removal of GR resulted in complete eradication after two consecutive years of removal; the SSB and population densities decreased by 99.34% and 99.79%, respectively. Nordby et al. (2005) stated that 95% GR seeds were lost from the top 20 cm of soil in conventional tillage and no‐tillage crop fields after two years when new additions to the seed bank were prevented. Goplen et al. (2017) reported that weed seed inputs in the cropping systems only needed to be prevented for two years to reduce the GR seed bank by 96%. Reducing the density of the SSB can be used as an effective and sustainable control method for annual invasive plants such as GR in grasslands. GR can grow even under repeated mowing, as the weed can produce many lateral branches as long as there is a sufficient growth period. This suggests that mowing once during GR reproductive growth in autumn could be an ideal solution for eliminating seed setting, yielding the benefits of effective good control and low cost.

Annual plants typically have a short life cycle, strong fecundity, and the ability to adapt to persistent and strong disturbance (Grime, 2001). Most annual invasive plants depend on the SSB to maintain their populations and to avoid extirpation. Therefore, the SSB density and germination of annual invasive plants determine their invasiveness and ability to spread. For example, techniques designed to reduce SSB density to control weeds have been used in farmland in the Midwest of the United States (Shaner & Beckie, 2014). For grasslands, cutting plants and removing seeds to reduce the SSB density have the advantages of being pollution‐free, sustainable, and less harmful to crop and pasture production. However, this requires a large amount of labor in the first 1–2 years, although the intensity of continuous treatment after three years is very low.

Methods designed to reduce SSB density have strong operational advantages in the early period of invasion. However, once a stable SSB is formed in the later period of invasion, the labor input and duration will be relatively prolonged. Even if the SSB density of GR was decreased by 99.34%, the SSB density of GR in the grassland remained at 31.9 grain m^−2^. Long‐term continuous treatment will be needed for effective control.

## CONFLICT OF INTEREST

None declared.

## AUTHOR CONTRIBUTION

**Hegan dong:** Conceptualization (lead); Data curation (lead); Formal analysis (lead); Funding acquisition (equal); Investigation (lead); Methodology (lead); Project administration (lead); Resources (lead); Software (lead); Supervision (lead); Validation (lead); Visualization (lead); Writing‐original draft (lead); Writing‐review & editing (lead). **Tong Liu:** Conceptualization (lead); Data curation (equal); Formal analysis (equal); Funding acquisition (lead); Investigation (equal); Methodology (lead); Project administration (lead); Resources (equal); Software (equal); Supervision (equal); Validation (equal); Visualization (equal); Writing‐original draft (equal); Writing‐review & editing (equal). **Zhongquan Liu:** Data curation (equal); Formal analysis (equal); Investigation (equal); Methodology (equal); Project administration (equal). **Zhanli Song:** Data curation (equal); Formal analysis (equal); Investigation (equal); Methodology (equal); Supervision (equal); Writing‐original draft (equal). 

## Data Availability

All raw data have been uploaded to Dryad with DOI accession number: https://doi.org/10.5061/dryad.6djh9w0x5.
